# Choledochal Cyst in Adults: Etiopathogenesis, Presentation, Management, and Outcome—Case Series and Review

**DOI:** 10.1155/2015/602591

**Published:** 2015-07-15

**Authors:** Norman Oneil Machado, Pradeep J. Chopra, Adil Al-Zadjali, Shahzad Younas

**Affiliations:** Department of Surgery, Sultan Qaboos University Hospital, P.O. Box 38, 123 Muscat, Oman

## Abstract

*Background*. Choledochal cyst, a rare congenital cystic dilatation of biliary tree, is uncommon in adults. Their presentations differ from children and surgical management has evolved.* Methods*. A retrospective review of the records of all the patients above 15 years, who underwent therapeutic intervention in our hospital, was carried out.* Results*. Ten cases of choledochal cyst were found; 8 female, with mean age 31 years. These included 8 cases of Todani type I and one case each of type II and type III. The predominant symptoms were abdominal pain and jaundice. Abdominal mass and past history of cholangitis and pancreatitis were seen in 2 patients. Investigations included ultrasound in 8 patients, CT in 7, ERCP in 3, and MRCP in 5. Surgical intervention included complete excision of the cyst with hepaticojejunostomy and cholecystectomy (type I), excision of the diverticulum (type II), and ERCP sphincterotomy (type III). Malignancy was not seen in any patients. The long-term postoperative complications included cholangitis in two patients.* Conclusion*. Choledochal cyst is rare in adults. The typical triad of abdominal pain, jaundice, and mass is uncommon in adults. The surgical strategy aims for single stage complete excision of the cyst with hepaticojejunostomy.

## 1. Introduction

**Figure 1:**
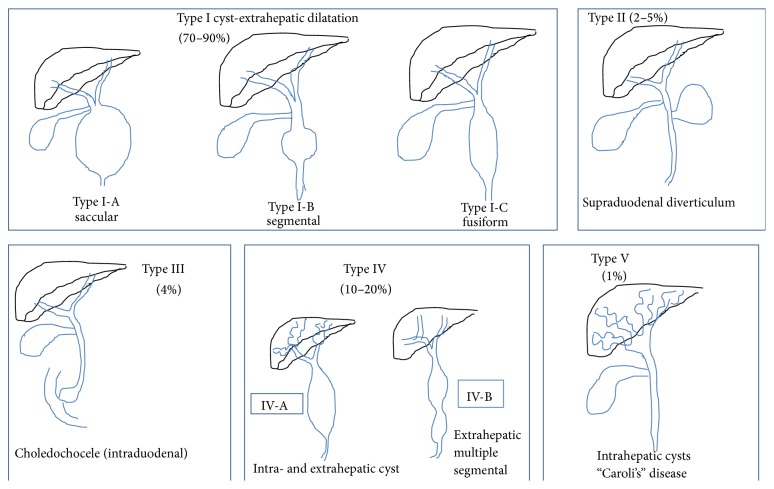
Todani's classification of choledochal cyst.

Choledochal malformation (CDM) is a pathological condition characterized by varying degree of congenital dilatation of the biliary system including the common, intrahepatic, and intrapancreatic bile duct. Presence of significant dilatation constitutes choledochal cyst (CCD). This entity occurs more frequently in Asia than in western countries with most reports originating from Japan [1–11]. The incidence ranges from 1 in 13,000 in Japan [1–3] to 1 in 2 million in England [4, 5]. However there are no reports of CCD in adults from Oman or Middle East, though a report of CCD in children is found [12]. They usually manifest in children and very few of them present when adults. About 25% of CCD is diagnosed antenatally or within the first year of life, 60% during the first decade of life and 20% go undiagnosed into adulthood [3–5]. A marked female preponderance has been widely recognized (female to male ratio 3 : 1) [3–9, 11, 13]. Presence of anomalous biliopancreatic duct junction (APBDJ) allowing pancreatic juice to reflux into biliary tree is the most widely accepted etiopathogenic concept [2–6, 11–15]. CCD is associated with biliary tree stasis and lithiasis and the whole biliary epithelium is considered at risk of malignant transformation [10, 14, 15]. Magnetic resonance cholangiopancreaticography (MRCP) is currently the most accurate preoperative imaging study to assess cyst anatomy and classify the disease according to standard Todani classification [3, 4, 10, 11] (Figure 1). Complete cyst excision with cholecystectomy followed by biliary reconstruction using Roux-en-Y hepaticojejunostomy is the treatment of choice for the extrahepatic component of the disease (type I and type IV CCD). In type V CCD (Caroli's disease), liver resection is tailored to the extent of intrahepatic disease and the presence and severity of underlying chronic liver (congenital hepatic fibrosis) and the associated kidney disease are taken into consideration [3, 4, 8]. In this report we present our experience with choledochal cyst and discuss the aetiopathogenesis, presentation, management, and outcome with review of the literature.

## 2. Material and Method

A retrospective review of the records of all the patients above 15 years who underwent excision of choledochal cyst or endoscopic intervention in our hospital in the period of 1998 and 2013 was carried out. Data regarding the clinical presentation, investigation, operation, and follow-up were analysed. The type of cyst was classified according to Todani classification.

## 3. Results

**Figure 2:**
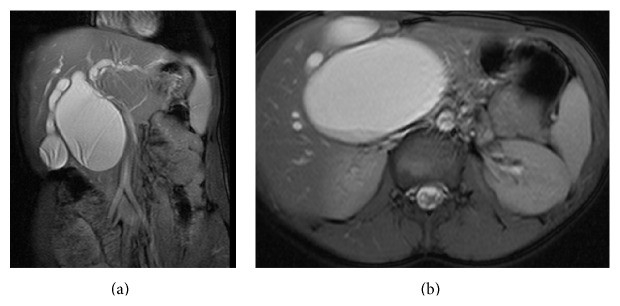
(a) MRCP showing type I-A saccular choledochal cyst in patient 4 (Table 1). (b) MRCP (cross section) showing large type I-A saccular choledochal cyst in patient 4 (Table 1).

Ten patients were treated for choledochal cyst, of whom eight were women. The median age of presentation was 31 years (16–38 years) and two of them were males with the mean age of 36 years (26 to 48 years). The predominant symptom was abdominal pain occurring in all patients. Two patients presented with history of recurrent cholangitis and another two of them presented with abdominal mass. The symptoms and complications at presentation are summarized in Table 1. The imaging studies carried out for diagnosis included abdominal ultrasonography in all ten patients, abdominal computed tomography (CT) in 7, ERCP in 3, and MRCP in 5 patients (Figures 2(a) and 2(b)). The cyst classification by Todani classification revealed eight cases of type I cyst (fusiform 5 and saccular 3) and one of each of type II and type III cysts (choledochocele).

**Figure 3:**
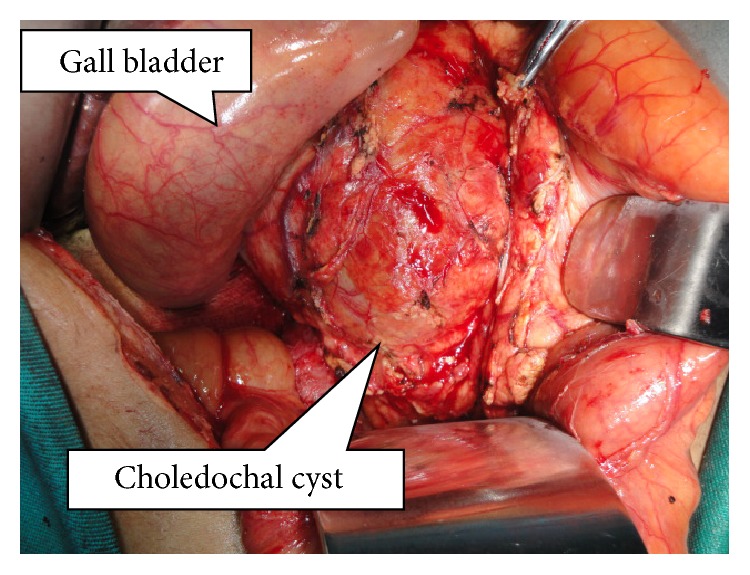
Intraoperative view of large choledochal cyst dissected out in patient 4 (Table 1).

**Figure 4:**
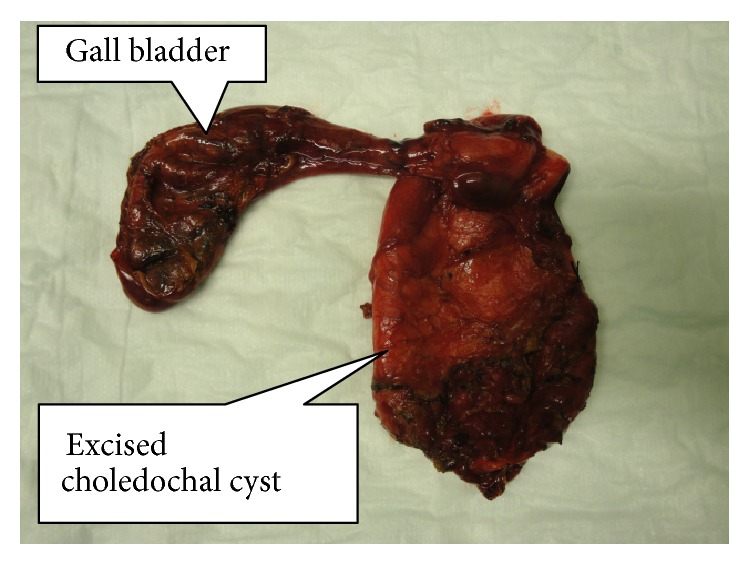
Resected specimen of completely excised choledochal cyst with gall bladder (patient 4).

**Figure 5:**
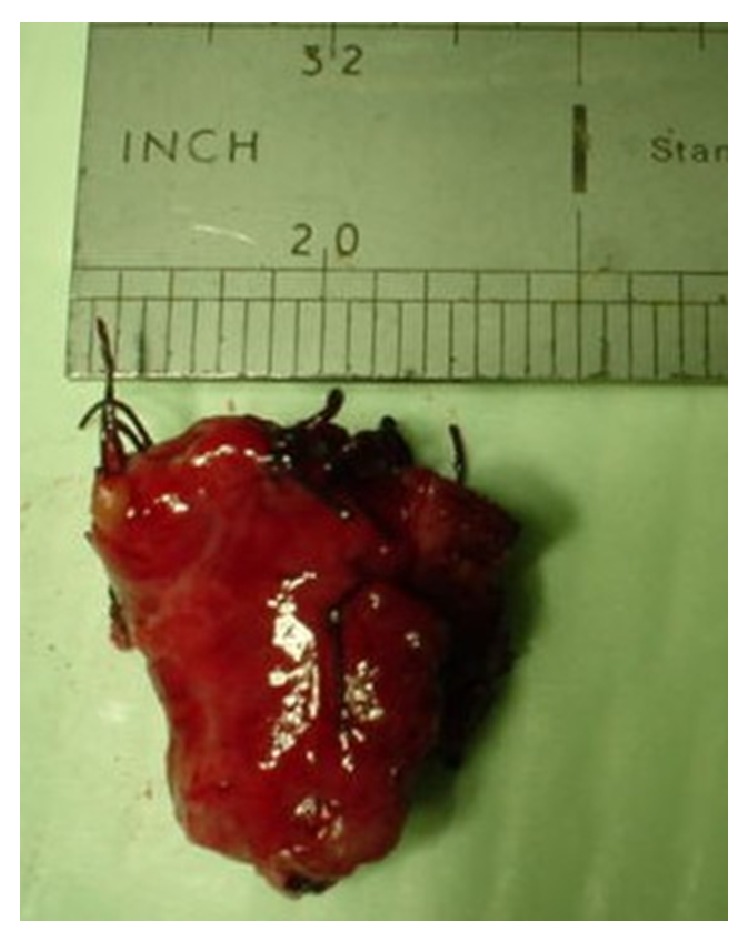
Complete excision of fusiform type I-C choledochal cyst in patient 2 (Table 1).

None of the patients had undergone preoperative drainage procedure. ERCP was carried out in 3 patients to define the APBDJ. The patient with choledochocele in addition underwent sphincterotomy. In none of these patients, malignancy was detected. One of the patients had undergone previous cystoduodenostomy (10 years back elsewhere, at the age of 18) and had recurrent cholangitis. Surgical strategy in eight patients with type I choledochal cyst consisted of complete cyst resection, cholecystectomy, and bilioenteric anastomosis (Figures 3, 4, and 5). In one patient with saccular type I cyst, the cyst was opened to define the transaction line of the upper end of the cyst. The operative procedure details are summarized in Table 1. The mean operating time was 2 hours and 40 minutes (1 hour 40 minutes to 4 hours range). Type II lesion was excised without any reconstruction and type III patient underwent ERCP sphincterotomy. All the resected specimens showed chronic inflammation. However, in one specimen in addition, features of mild dysplasia were noted.

The early outcome was that one of the patients developed mild pancreatitis and anastomotic leak, which was managed conservatively with IV fluids, antibiotics, and ultrasound guided drainage. The median duration of hospital stay was 10 days (range 3–32). Patients were followed up for a median duration of 6 years (range 3 months to 12 years). There was no perioperative mortality. All patients were symptom-free, except for two patients who developed 2 episodes of recurrent cholangitis during their follow-up and were managed successfully with antibiotics. No anastomotic strictures or malignancy was noted in any of these patients during follow-up. In addition, no patients developed nutritional abnormalities and they were in good health during the period of follow-up.

## 4. Discussion

**Figure 6:**
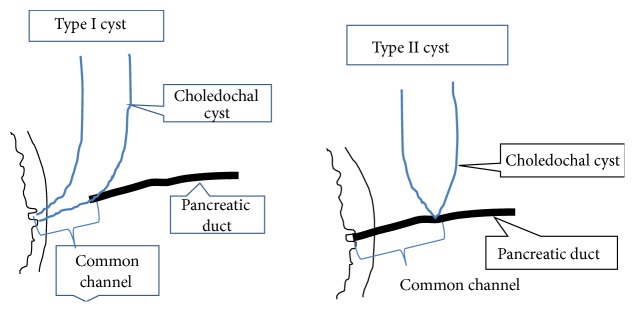
Types of abnormal union of pancreaticobiliary junction. Type I cyst (PC junction)—main pancreatic duct joins the common bile duct. Type II cyst (CP junction)—the common bile duct enters the main pancreatic duct.

Choledochal malformation (CDM) is characterized by dilatation of the biliary tract in the absence of acute obstruction to the bile flow [16]. Those of these malformations with cystic dilatation constitute the choledochal cyst [16]. This is rare entity and was first described by Vater and Ezler [17]. Cystic dilatation may occur in any part of the bile duct from liver to the duodenum. CCD is classified according to the site (extrahepatic or intrahepatic), extent (segmental or complete), and shape (cystic, saccular, and fusiform). The Todani classification classifies choledochal cyst into 5 major types [7] (Figure 1). The etiology of CCD remains unclear. However, there are 2 commonly proposed hypotheses to explain this. The simplest of this relates this to a partial obstruction of the bile duct, leading to increased proximal bile duct pressure and eventual dilatation, initially of the extrahepatic segment and subsequently the intrahepatic component [16]. The second theory known as Babbitt's hypothesis is based on the pathophysiological consequence of reflux of activated proteolytic pancreatic enzymes on the biliary tract wall [18]. The wall eventually is believed to undergo weakening due to prolonged exposure to activated proteolytic enzymes, leading to cystic dilatation. The abnormal communication predisposes to reflux of pancreatic juice into bile duct, leading to ineffective bile flow which in turn results in increased intraductal pressure, chronic inflammation, and its associated carcinogenic effect [14, 17, 19]. This reflux occurs due to the existence of common channel of biliary and pancreatic juice drainage. Several reports suggest a close association of CCD with anomalous union of the pancreaticobiliary duct (APBDJ) [14, 17, 20]. This results in a common channel of >15 mm and the abnormalities are classified into 2 types, choledochal-pancreatico or pancreatico-choledochal junction, and may influence the degree of choledochal dilatation [21] (Figure 6). The prevalence of long common channel ranges from 96 to 100% in paediatric series [5, 20] and from 68% to 94% in adult series [2–7]. However, a recent report studied the relationship of choledochal pressure, bile amylase activity (indicating reflux), and morphology of the choledochus. High choledochal pressure and not the bile amylase level was found to be associated with more severe histopathological changes and choledochal morphology. They inferred that the distal bile duct obstruction and hence the high intraluminal pressure contribute predominately to the key features of choledochal malformation rather than pancreatic reflux [16]. In addition to the above hypotheses, the literature reveals several other factors postulated to contribute to the development of CCD. These include obstruction to the bile duct due to sphincter of Oddi dysfunction, inadequate autonomic innervation, atresia, stenosis, and fibrosis of the terminal bile duct [20, 22]. These abnormalities however are often not easily identifiable [5]. The origin of CCD may be as a consequence of wider spectrum of these pancreaticobiliary disorders and it is likely that different pathogenetic mechanism is probably responsible for the different cyst types observed in adults and children [5, 6, 14].

Caroli's disease is a rare and complex autosomal congenital disorder that presents as cystic dilatation of the intrahepatic bile ducts. The main mode of Caroli's disease inheritance is autosomal recessive type. Mutations in the polycystic kidney and hepatic disease gene 1 (*PKHD1*) are responsible for this condition. However Caroli's disease does not start with dilatation and fibrosis is a late event [23]. Embryological malformation of the ductal plate may lead to abnormal bile ductular proliferation and configuration contributing to type V CCD. Involvement of large intrahepatic ducts leads to Caroli's disease. Whereas diseased small interlobular bile ducts result in congenital hepatic fibrosis, involvement of all levels of biliary tree results in both congenital hepatic fibrosis and Caroli's disease. The term “Caroli's syndrome” is used when Caroli's disease is associated with kidney disease (from tubular ectasia to polycystic kidney disease) [24].

Children and adults with CCD often have different signs and symptoms [3, 5, 10, 25]. The classical triad of jaundice, right hypochondriac pain, and a palpable mass was found more commonly in children compared to adults (85% versus 25%, resp.) whereas abdominal pain, cholangitis, pancreatitis, and history of cholecystectomy for biliary symptoms were more common in adults [10, 11, 15, 25] as was observed in our patients (Table 1). CCD may remain asymptomatic for many years and diagnosis may be made incidentally, when asymptomatic patients undergo imaging studies for unrelated process; however, these patients could present for the first time with complicated clinical presentation, including cholangitis, liver abscess, and biliary cirrhosis, and this is more likely to occur in adults than in children [2–7, 10–13]. A lag period of 6 years is often noted particularly in adults between the development of symptoms and diagnosis and treatment [10, 11].

CCD may be diagnosed in asymptomatic patients undergoing health screening test, when liver function test (LFT) is found to be abnormal. In one of the reports, 9% of the patients with abnormal LFT during health screening test were detected to have CCD, all of whom were otherwise asymptomatic [3]. There has been an evolution in what is regarded as best imaging method for diagnosing and assessing choledochal cyst ranging from abdominal ultrasound initially to MRCP presently [2–7, 10–13]. CCD can now be diagnosed at any age of life including antenatally by ultrasonography [11]. Precise preoperative identification of the type, extent of biliary tree dilatation, and information on the pancreatic and bile duct anatomy and disease are essential to plan surgical strategy [2–7, 10–13]. While ultrasound may be favoured as an initial investigation in assessing the choledochal cyst due to the ease of performing it, its limitations may be in differentiating choledochal cyst from gall bladder distension due to cholecystitis [3, 11]. This is reflected by large percentage of adult patients with choledochal cyst being identified for the first time during cholecystectomy, indicating that ultrasonography study may underestimate the diagnosis [11]. The choledochal cyst may have been missed on ultrasonography because of technical quality of the examination or a failure to recognize an uncommon pathology [3, 10, 11]. However if choledochal cyst is suspected, then ultrasound is usually diagnostic. Computed tomography provides important information about the extrahepatic or intrahepatic extent of biliary dilatation [3, 10, 11].

The current “gold standard” for staging CCD is magnetic resonance cholangiopancreaticography (MRCP) [4, 10, 11, 15] (Figures 2(a) and 2(b)). MRCP has the distinct advantage of being noninvasive in nature and in its ability to assess cyst anatomy, identify size, site, and shape of bile duct dilatation, and detect APBDJ making it distinctly superior [26]. It also avoids the risk of potential complications of pancreatitis and cholangitis associated with invasive procedures like ERCP and percutaneous cholangiography. MRCP also facilitates the reliable diagnosis of Caroli's disease based on finding of cystic intrahepatic cavities communicating with intrahepatic biliary tree [27]. Gadoxetic acid enhanced MRCP can visualize the physiology of bile excretion, in contrast to conventional T2-weighted and fat suppression images. Gadoxetic acid is taken up by hepatocytes and excreted into the bile duct that allows visualization of the bile ducts on hepatobiliary phase of T1-weighted images [28]. However, in cases where there are limitations in performing MRCP as in those with the possibility of artifacts due to intra-abdominal clips from previous surgery, claustrophobia or unavailability of facility and then cholangiogram can be considered. Cholangiography will help in differentiating the type of CCD and in planning the extent of resection, in case MRCP facility is not available. ERCP best visualizes the pancreaticobiliary junction but may not define the superior intrahepatic extent of the cyst, if cysts are redundant and sequestrate large amount of contrast material [3, 4, 10–13, 15]. Preoperative transhepatic cholangiography has been preferred by some because of its ability to define the proximal extent of biliary dilatation facilitating preoperative planning for resection [3–6, 13, 15]. Cytology of bile ducts specimen taken during ERCP or a PTC by brush or needle biopsy plays an additional role in cholangiocarcinoma diagnosis. Even though a negative cytology from brushings does not exclude malignancy, combined brush and biopsy cytology specimen increases sensitivity to 40%–70% [28].

The complications associated with CCD include stone formation secondary to bile stasis in the cyst and intrahepatic ducts, recurrent cholangitis, pancreatitis, and spontaneous cyst rupture due to raised intra-abdominal pressure as in pregnancy [2–7, 10–15, 27, 29]. In addition, the coexistent congenital hepatic fibrosis in patients with type V CCD predisposes to portal hypertension and oesophageal varices [2, 5, 7, 8]. The reported incidence of cholelithiasis due to bile stasis is around 37.5 to 74% [26, 27]. Hepatolithiasis is most often noted in type IV-A CCD and may be related to the presence of membranous or septal stenosis or segmental bile duct near main biliary convergence [30]. The abnormal pancreaticobiliary junction in the presence of obstruction by stones or protein plug impaction predisposes to the risk of acute pancreatitis, which is reported to be seen in 30–70% of adults [10, 11].

However the major concern is the risk of malignant transformation, which is well documented in the literature [1–15]. The whole biliary tree is considered at risk of malignant transformation and may arise either in cystic dilatation or remnant tissues after excision or in nondilated parts of the biliary tree including the gall bladder. The age at diagnosis of CCD is related to the development of carcinoma in the gall bladder, the cyst, or the intrahepatic ducts. In patients who have CCD at 10 years of age or younger, the risk of developing cholangiocarcinoma is approximately 1%, whereas the risk increases to 15% for patients older than 20 years of age, 26% in patients above 40 years, and 45.5% in patients above 70 years [13, 28]. The incidence of synchronous cholangiocarcinoma associated with CCD is estimated to be 2.5 to 30% and was 6% in the largest reported western series [3–5, 10–13, 15]. Todani et al. collected data from 73 institutions in Japan and reported an incidence of 17.5% that is higher than 0.01 to 0.38% incidences found in large autopsy series in normal population [29]. The histological types of cancer are adenocarcinoma (73%–84%), anaplastic carcinoma (10%), undifferentiated cancer (5%–7%), squamous cell carcinoma (5%), and others (1.5%) [31]. The locations of the cancer are extrahepatic bile ducts (50–62%), gall bladder (38%–46%), intrahepatic bile ducts (2.5%), liver (0.7%), and pancreas (0.7%) [31].

Surgery for CCD has evolved, both in the timing of surgery and in the type of surgery carried out [1–15, 18–21]. The current approach to CCD involves control of biliary sepsis and pancreatitis and defining both the superior and inferior extent of the cyst, before scheduling surgery in semielective setting [2–7]. Inadequately prepared patient may lead to technically difficult operative field due to adhesions to adjoining structures. Moreover, choice of surgery may be inappropriate due to inadequate preoperative assessment of the type and extent of CCD or misinterpretation of preoperative imaging, whereby the nature and extent of CCD are only discovered intraoperatively [1–7, 10–13].

The main objective of surgical intervention is an attempt for complete excision of the cysts to avoid long-term consequences of cholangitis, liver cirrhosis, pancreatitis, and malignant transformation [1–7, 10–13]. These problems can be exacerbated if an internal drainage procedure (cystoduodenostomy or cystojejunostomy) is performed (as in one of our patients) rather than a cyst resection [4–6]. Palliative procedures are indicated only when the associated comorbidities and the general fitness of the patient are not conducive for resection [4–6]. Complete excision of extrahepatic component of CCD combined with cholecystectomy, followed by Roux-en-Y biliary reconstruction, is considered to be the treatment of choice for type I and IV CCD [1–7, 10–18]. Cholecystectomy is carried out due to the high risk of associated gall bladder malignancy, particularly in patients with APBDJ [13, 18]. Although all portions of CCD should be removed, residual proximal cyst wall may be left by some to facilitate biliary anastomosis [3–6, 10, 11, 13]. Complete cyst excision requires accurate recognition of the origin and termination of cyst [5, 6]. The ends of cysts extending from the confluence of the hepatic duct to the junction of the common duct and the pancreatic duct are very difficult to define clearly [3–6, 10, 11, 13]. Difficulty is also encountered in differentiating the normal bile duct endothelium from the cyst lining, by means of intraoperative frozen section biopsies [3]. Some opt for the technique of inspecting the luminal appearance grossly, after opening the cyst to determine the proximal transaction line in healthy biliary tissue [3]. This was done in one of our patients. The exact level of anastomosis is a balance between the need for complete cyst resection and the need to achieve widely patent anastomosis [3–6, 10, 11, 13, 14]. If the hepatic duct opening appears smaller or hypoplastic, then a wide anastomosis may be beneficial, after leaving some cyst wall remnant [2–6]. Biliary reconstruction is performed with a long defunctionalized Roux limb anastomosed to the transected common hepatic duct or more frequently at the upper biliary convergence after opening the hepatic ducts [2–6, 10, 15].

Posteriorly the cyst wall is generally easy to free from the portal vein and hepatic artery [2–6]. However complete excision may be difficult with a history of recurrent cholangitis and marked adhesion to surrounding tissues. In such patients only the anterolateral aspect of cyst is excised followed by resection of mucosal lining of the back of the cyst adjoining the portal vein and the hepatic artery [2–6, 10, 13, 15]. This is achieved by carefully dissecting with electrocautery leaving a rim of the posterior cyst wall (Lilly's procedure) [32]. Dissection of the intrapancreatic cyst is exposed to the potential risk of pancreatic duct injury [3–6, 10, 11, 13]. Several methods have been employed to define the junction including intraoperative endoscopy and ultrasound and intraoperative cholangiography, after the placement of hemoclips [4, 24, 33, 34]. If the cystic lesion occupies most of the pancreatic head and if there is no noticeable distance (less than 5 mm) between the cyst neck and pancreaticobiliary junction, then pancreaticoduodenectomy may be considered. However, the decision is made only after assessing whether the risk of leaving some intrapancreatic cystic remnant outweighs the risk of surgery and the long-term nutritional disadvantages of pancreaticoduodenectomy [3–6, 10, 11, 13, 14]. If a cystic portion must be left behind, obliteration of the cyst lumen is advisable to prevent stasis of the pancreatic juice [3–6]. This is achieved in large cystic dilatation by transecting the cyst wall few cms proximal to the head of pancreas leaving an “egg-cup bottom” which is then removed by intraluminal dissection [5]. This technique reduces the risk of bleeding and pancreatic duct injury [5].

Following the cyst excision the hepaticoenterostomy can be carried out by 2 types of anastomosis: hepaticoduodenostomy or Roux-Y hepaticojejunostomy [35]. In general, the success of an anastomosis is measured by the ease of performing it and the short- and long-term complications. The reported success of hepaticojejunostomy is 92% with complication rate of 7% compared with complication rate of 42% following hepaticoduodenostomy [35, 36]. Hepaticoduodenostomy is not recommended by some, because of the reported complications (33.3%), which include bilious gastritis due to duodenogastric bile reflux and adhesive bowel obstruction and cholangitis. In addition, increased risk of gastric cancer (due to bile reflux) and biliary cancer has been reported [36]. On the other hand, there are others who are proponents of hepaticoduodenostomy because of its simplicity, being quicker to perform, and importantly preservation of normal anatomy and physiology and minimum complications [37].

Type II CCD is managed by complete but limited cyst excision [3–6, 10, 11, 13, 15, 20, 21]. Extrahepatic bile duct resection may only be necessary in case of a large neck of the cyst at its junction with the common bile duct and is not routinely recommended. In the presence of APBDJ, the patient is exposed to the risk of malignancy in both the gall bladder and bile duct, and a prophylactic excision of gall bladder is advised [2–6]. In view of the rare risk of malignancy in patients with type III CCD, transduodenal cyst excision is currently replaced by conservative endoscopic sphincterotomy as treatment of choice, particularly when the cyst is less than 2 cms in diameter. Pancreaticoduodenectomy is carried out in the presence of coexistent cancer [2–6, 10, 15].

Type IV-A and type V cysts are unique in that the cystic dilatation extends to the intrahepatic biliary tree [2–11, 13, 38–42]. Total cyst excision is often impossible and a different approach is necessary as the management remains controversial [2–11, 13]. While the extrahepatic component in type IV-A and type B cysts is treated by excision, the intrahepatic component is addressed differently. There are some who would consider resection of the segment of lobes affected by hepatolithiasis, hepatic abscess, and ductal strictures, while there are others who would consider a more conservative approach with preservation of hepatic parenchyma even in the presence of hepatic calculi and strictures, provided the liver is not cirrhotic [2–6, 10, 11, 15, 38, 41, 42]. Such patients are treated with placement of large bore silastic transhepatic stents to facilitate postoperative stone extraction [29]. Liver transplantation may offer a more durable solution and has to be offered in case of diffuse intrahepatic disease complicated by intrahepatic calculi, recurrent cholangitis refractory to medical treatment and in those with secondary cirrhosis [2–6, 10, 11, 13, 15, 43].

The management of type V CCD is particularly difficult and poses special problems [2–8]. The disease is complicated by the extent of the disease in the liver (localized or diffuse) and it is often association with congenital hepatic fibrosis, secondary biliary cirrhosis, and kidney disease [2–6, 10, 15]. The condition is dealt with drainage procedures, which are palliative in nature and run the risk of being ineffective in the long term and are associated with recurrent cholangitis. When the disease is localized to unilobar intrahepatic disease without associated chronic liver disease, liver resection is the optimal choice [41, 42]. However when the disease is more diffuse involving both lobes or associated with portal hypertension from congenital hepatic fibrosis or secondary biliary cirrhosis, then these are appropriate candidates for liver transplantation [41, 42]. The long-term outcome is better when transplantation is carried out at an earlier stage of the disease, avoiding numerous ineffective operative procedures and an emergency operation in septic patients [2–4, 10, 44].

The management of concurrent malignancy in the biliary tract/cyst in patients with CCD will be along the same principle applied for malignancy in patients without CCD [4, 10, 28]. Patients with distal malignancy will undergo pancreaticoduodenectomy with standard clearance of regional lymph nodes. Those involving the proximal biliary tract would be managed as in patients with Klatskin tumour. In patients with type I and II lesion, an en bloc resection of the extrahepatic bile ducts and gall bladder, with regional lymphadenectomy and Roux-en-Y hepaticojejunostomy, would be performed [28]. Those with type III lesion would require extended right or left hepatectomy, in addition to the above procedure. Type IV lesions would be dealt with with extended right or left hepatectomy, in addition to the above procedure [28]. The intrahepatic segment malignancy would require resection of the involved segments or lobes of the liver [28].

Laparoscopic choledochal cyst excision and hepaticojejunostomy for type I and type II cyst have been described as an alternative to open procedure [43, 45]. This option is reported to be particularly attractive in paediatric patients owing to its advantage of reduced intraoperative stress, faster recovery, and superior cosmetic result [43, 45]. Unlike in adults, the cysts are at less risk of being scarred due to recurrent episodes of cholangitis leading to relatively easier laparoscopic dissection and shorter operating time. However, in general, the distinct disadvantage of laparoscopic approach is that the complete excision with hepaticojejunostomy is technically challenging and time consuming, with a reported conversion rate of 10–37% [43, 45]. In a recent review comparing the outcome of open versus laparoscopic approach, the overall complications and its degree were comparable (grades I-II, *n* = 13, and grades III-IV, *n* = 5, versus grades I-II, *n* = 5, and grades III-IV, *n* = 5). The overall 5-year survival rate was also similar being 98% versus 100%, respectively [46]. The outcome of laparoscopic versus open surgery was recently reviewed in a meta-analysis involving a large number of children with CCD [47]. Studying 1016 patients of whom 408 patients underwent laparoscopic cyst excision and Roux-en-Y hepaticojejunostomy (LH) and 608 cases underwent open cyst excision and Roux-en-Y hepaticojejunostomy (OH), the following observations were made [46]. The patient undergoing LH had longer operative time (MD = 59.11, 95% CI 27.61–90.61, *P* = 0.0002), while the length of postoperative hospital stay was shorter (MD = −2.01, 95% CI–2.49 to −1.54, *P* ≤ 0.00001), intraoperative blood loss was lower (MD = −37.14, 95% CI −66.69 to −7.60, *P* = 0.01), and food intake was earlier (MD = −1.14, 95% CI −1.61 to −0.67, *P* = 0.01) [47]. Moreover the postoperative morbidity was found to be more in the OH group, though not statistically significant [46].

## 5. Outcome

All patients postoperatively will require lifelong surveillance for malignancy as there is a 20- to 30-fold increased risk of developing cholangiocarcinoma, compared with the general population. About 2.5 to 30% will eventually develop cholangiocarcinoma [2–7, 10, 11, 13, 20, 22]. Women are more commonly affected, a reflection of the increased prevalence of choledochal cyst in female patients with increasing risk as age advances [3, 6, 15]. Metachronous carcinoma can develop throughout the biliary tree in nondilated intrahepatic bile ducts, at the anastomotic level or in the distal intrapancreatic common bile duct [3–6, 10, 11, 13, 15]. The risk of malignancy persists even after cyst excision, but the risk is higher for patients in whom residual cyst is left behind [3–6]. Closer surveillance is indicated for patients with type IV cysts and in patients with APBDJ, as the incidence of malignancy is higher in these cases [3–6, 10, 11, 13, 14]. All patients with CCD require long-term follow-up for bile duct cancer, using ultrasonography and laboratory investigations including liver function parameters and tumour markers (CEA, CA 19-9, and CA-125) [35]. CA 19-9 is the most significant because it is elevated in up to 85% of patients with cholangiocarcinoma. CEA is raised in about 30% of the patients and CA-125 in 40%–50% of patients with cholangiocarcinoma [28]. The other delayed postoperative complications include cholangitis, intrahepatic strictures, and/or lithiasis or strictures occurring in most cases at anastomotic stoma [3–6]. The predisposing factors for anastomotic stricture include type IV-A CCD, large cystic size, shorter duration of symptoms and increased infiltration of inflammatory cells, inadequate blood supply to the bile duct stump, and the size of the anastomosis [46]. Late occurrence of anastomotic stricture and intrahepatic duct stones are reported in 23.5% of cases and are significantly more frequent in type IV-A cyst, justifying the need for careful long-term follow-up in them [47]. Anastomotic stricture complicating cyst excision can be treated by surgical revision or by percutaneous transhepatic dilatation [11, 48, 49]. Treatment of hepatolithiasis by transhepatic endoscopic lithotripsy and percutaneous stones removal requires repeated hospital admissions and treatment courses [11]. Percutaneous stone removal may be facilitated by placement of large silastic transhepatic stents at the time of primary surgery [2–6, 10, 11]. Creation of jejunal loop attached to the anterior abdominal wall leading to hepaticojejunostomy would make the treatment easier by approaching the intrahepatic ducts through a percutaneous anterior abdominal transjejunal route [3, 10]. Others have proposed a wider hepaticoduodenostomy at the biliary convergence to assess intrahepatic biliary tree endoscopically during follow-up [4–6, 10].

## 6. Conclusion

CCD is a rare congenital abnormality in adults. A better understanding of the natural history of CCD has allowed surgeons to tailor the management accordingly. Emphasis should be placed on thorough preoperative assessment of cyst anatomy and the necessary limits of resection. This to a large extent is achieved by imaging methods like MRCP and ERCP preoperatively and by on-table cholangiography and endoscopic ultrasound intraoperatively. Abnormal APBDJ is likely to be seen in the paediatric patients more than in adults. Abdominal mass and jaundice are common presentation in children, while the adult patients are more likely to present with complications including cholangitis, choledocholithiasis, pancreatitis, and malignant transformation. Complete cyst excision is the treatment of choice for extrahepatic component of the disease, although the optimal treatment of intrahepatic bile duct dilatations remains controversial, especially for type IV-A CCD. Liver transplantation as an option is most suited for patients with diffuse intrahepatic form of the disease complicated by stones, recurrent cholangitis, fibrosis, cirrhosis, and portal hypertension. For type V (Caroli's disease), the extent of liver resection is tailored to that of intrahepatic disease and takes into consideration the presence and severity of underlying chronic liver (congenital hepatic fibrosis) and kidney disease. Due to the age related risk of synchronous and metachronous cholangiocarcinoma, complete cyst excision should be carried out early. Long-term follow-up is required in these patients as they are prone to cholangitis, anastomotic stricture, and malignancy in the residual biliary tree.

## Figures and Tables

**Table 1:** Demographic details, management, and outcome of patients.

Pt	Age/sex	Symptoms/signs	Type of cyst	Previous surgery	Investigations	Procedure	Postop comp. early	Postop comp. late	Hospital stay (days)
1	38/F	Abd. pain, acute pancreatitis	Type III	Nil	US, CT, and ERCP	ERCP sphct	Nil		3

2	28/F	Abd. pain, Jn, and cholangitis	Type I-C	Choledochoduodenostomy	US/CT/MRCP	CC/CEx/HJ	Nil		10

3	32/F	Abd. pain, Jn	Type I-A	Nil	US/ERCP/MRCP	CC/CEx/HJ	Nil		10

4	16/F	Abd. pain, Jn, cholangitis, and abd. mass	Type I-A	Nil	US/ERCP/MRCP	CC/CEx/HJ	Mild pancreatitis/ anastomosis leak	Chol.	32

5	22/M	Abd. pain	Type II	Nil	US/MRCP	Excision	Nil		7

6	37/M	Abd. pain, Jn, and acute pancreatitis	Type I-A	Nil	US/CT	CC/CEx/HJ	Nil		7

7	38/F	Abd. pain, Jn, and abd. mass	Type I-C	Nil	US/CT	CC/CEx/HJ	Nil	Chol.	8

8	30/F	Abd. pain	Type I-A	Nil	US/CT	CC/CEx/HJ	Nil		8

9	25/F	Abd. pain, Jn	Type I-A	Nil	US/CT/MRCP	CC/CEx/HJ	Nil		7

10	29/F	Abd. pain, Jn	Type I-C	Nil	US/CT	CC/CEx/HJ	Nil		7

Pt = patient number, abd. = abdominal, Jn = jaundice, US = ultrasound, MRCP = magnetic resonance cholangiography, ERCP = endoscopic retrograde cholangiopancreatography, CC = cholecystectomy, CEx = complete excision of cyst, HJ = hepaticojejunostomy, sphct = sphincterotomy, comp. = complications, chol. = cholangitis.
